# *MICA* Polymorphism and Genetic Predisposition to T1D in Jordanian Patients: A Case-Control Study

**DOI:** 10.3390/life12111813

**Published:** 2022-11-07

**Authors:** Wassan Jarrar, Sawsan I. Khdair, Feras A. Khudeir

**Affiliations:** 1 Department of Pharmacy, Faculty of Pharmacy, Al-Zaytoonah University of Jordan, Amman 11733, Jordan; 2 Jordanian Royal Medical Services, Amman 11855, Jordan

**Keywords:** type 1 diabetes, *MICA*, polymorphism, genetic risk, NKG2D, Jordan

## Abstract

Type 1 diabetes (T1D) is an autoimmune disorder whose etiology includes genetic and environmental factors. The non-classical Major Histocompatibility Complex (MHC) class I chain-related gene A (*MICA)* gene has been associated with increased susceptibility to T1D as the interaction of MICA to the Natural Killer Group 2D (NK2GD) receptors found on the cell surface of natural killer (NK) cells and T cells is responsible for inducing immune responses. *MICA* polymorphisms were reported in association with T1D among different ethnic groups. However, data from different populations revealed conflicting results, so the association of *MICA* polymorphisms with predisposition to T1D remains uncertain. The aim of this sequencing-based study was to identify, for the first time, the possible *MICA* alleles and/or genotypes that could be associated with T1D susceptibility in the Jordanian population. Polymorphisms in exons 2–4 and the short tandem repeats (STR) in exon 5 of the highly polymorphic *MICA* gene were analyzed. No evidence for association between T1D and *MICA* alleles/genotypes was found in this study, except for the MICA*011 allele which was found to be negatively associated with T1D (*p* = 0.023, OR = 0.125). In conclusion, *MICA* polymorphisms seem not to be associated with increasing T1D susceptibility in Jordanian patients.

## 1. Introduction

Type 1 diabetes (T1D) is an autoimmune disease which results from autoreactive immune cells destructing pancreatic β-cells [1]. T1D incidence among the world varies drastically and its prevalence in Arab countries is relatively high [2]. As far as we know, the prevalence and incidence of T1D in Jordan remains underreported. Development of T1D is induced by the complex interaction of several susceptibility genes in addition to environmental factors [1]. Human leukocyte antigen (*HLA*) genes play a pivotal role in autoimmune diseases [3]. In particular, the major genetical risk for developing T1D is conferred by *HLA class II* genes as individuals with the *DR3-DQ2/DR4-DQ8* haplotypes were shown to have the highest risk of developing T1D in different ethnic populations even in Jordanian T1D patients [1,4,5,6,7]. Yet, numerous studies suggest a possible role of other genes which are included in the *HLA* region in predisposition to T1D [8,9,10,11,12]. Particularly, the non-classical Major Histocompatibility Complex (MHC) class I chain-related gene A (*MICA*) has been associated with increasing predisposition to T1D [13,14,15,16,17,18,19]. *MICA* gene is considered one of the functional *HLA* class I genes [20]. *MICA* gene encodes a protein that is basically expressed in endothelial cells, gastrointestinal epithelium, as well as fibroblasts and whose expression is induced under stress conditions [21,22,23]. MICA protein serves as a ligand of Natural Killer Group 2D (NKG2D) receptors, which are found on the surface of natural killer (NK) as well as CD8^+^ T cells leading to the activation of these cells which are known to play a major role in the pathogenesis of T1D [22,24,25,26,27,28,29]. *MICA* is composed of six exons and five introns and is considered the most polymorphic non-classical class I gene [21]. Actually, 198 alleles of the human *MICA* gene have been recognized thus far according to the IMGT/HLA database; June 2022 [30,31]. There are several genetic variations within exons 2–4 of the *MICA* gene which encode the three extracellular domains of the MICA protein (α1, α2, and α3) which are recognizable by the NKG2D receptor. Accordingly, studies that analyzed the binding sites of MICA and NKG2D receptor suggest that the polymorphic sites located in exons 2–4 might influence the binding of MICA to its receptor [32,33]. In addition, the transmembrane region (TM) of the MICA protein, which is encoded by exon 5 of the *MICA* gene, was also found to be highly polymorphic. This so-called trinucleotide repeat microsatellite polymorphism (GCT)n consists of eight alleles; *A4, A5, A6, A7, A8, A9, A10,* and *A5.1*, and the names of these alleles were designated according to the number of repetitions of GCT nucleotides that these alleles possess [20,21,34,35,36,37,38].

To ascertain the relation of *MICA* with the predisposition to T1D, association studies were performed to determine T1D-related *MICA* alleles among different ethnic groups. However, data from different ethnic groups revealed controversial results [13,14,15,16,17,39,40,41,42,43]. To date, *MICA* alleles and genotypes that play a role in T1D susceptibility in the Jordanian population remain to be determined. Therefore, the aim of this study was to investigate for the first time the association of specific *MICA* alleles and genotypes with susceptibility to T1D in the Jordanian population taking into consideration both the polymorphisms in exons 2–4 as well as the short tandem repeats (STR) located in exon 5 of the *MICA* gene.

## 2. Materials and Methods

### 2.1. Study Population

This study included 106 Jordanian participants: 51 healthy unrelated volunteers and 55 T1D patients (Pediatric Endocrinology department of King Abdullah University Hospital). Clinical parameters of the volunteers are listed in Table 1. Regarding gender distribution, both studied groups were not fully matched. However, no previous studies reported gender differences in regard to *MICA* allele association [13,14,15,16,40]. Inclusion criteria for T1D patients in this study were a clinical onset of the disease before 18 years of age as well as a diagnosis based on the criteria of American Diabetes Association (ADA) guidelines. Moreover, the mean age of disease onset in T1D patients was 10.1 ± 7.4 years. Furthermore, healthy volunteers with a family history of diabetes were not included in this study and all controls were above 20 years old [44]. All participants were of Jordanian descent. Ethical approval (IRB approval number: 46/117/2018) was provided by the institutional review board of King Abdullah University Hospital. An informed consent was signed prior to enrollment, in accordance with the declaration of Helsinki [45].

### 2.2. DNA Extraction and MICA Genotyping

DNA was isolated from blood as described previously [46]. Typing of exons 2–4 as well as exon 5 of the *MICA* gene was performed using amplicon sequencing (Miseq Illumina platform). Primers designed to amplify exons 2 to 4 as well as exon 5 of the *MICA* gene are summarized in Table 2. After amplifying regions of interest in the *MICA* gene, next-generation sequencing (NGS) was completed by Genochem World SL. (Valencia, Spain). Capillary electrophoresis using the QIAxcel Advanced System (Qiagen, Barcelona, Spain) was performed to evaluate the quality of PCR products and the MiSeq Reagent Kit v2 (500-cycle) was utilized for sequencing.

### 2.3. Determination of Allele Frequency and Statistical Analysis

Direct counting was used to determine allele frequencies. *MICA* alleles and genotypes frequencies among T1D and control groups were compared using chi-squared test (χ^2^). The *p* value was considered significant when *p* value ≤ 0.05. Odds ratio (OR) calculation was performed as described previously [47] with a confidence interval (CI) of 95%. We conducted statistical analysis using the SPSS program (IBM analytics, Armonk, NY, USA).

## 3. Results

Sequencing results of exons 2–4 of the *MICA* gene revealed the presence of 24 different *MICA* alleles among the study group (both control and T1D groups) (Table 3). The most prevalent allele in the T1D patients’ group was MICA*009 with a frequency of 23.6%, followed by MICA*008, MICA*002, and MICA*004 with a frequency of 13.6%, 13.6%, and 10.9%, respectively. Nevertheless, the frequencies of most alleles in the T1D patient group were found to be comparable, with no significant differences, to the healthy control group. However, the only allele with a significant difference (*p* = 0.023, OR = 0.125, CI = 0.015–1.030) in frequency between the control and T1D patient group was MICA*011, with a frequency of 6.9% in controls and a lower allelic frequency in T1D patients’ group (0.9%).

A wide variety of genotypes (55 different genotypes) have been identified among the study groups (Table 4). The genotype with the highest frequency in the T1D patients’ group was MICA*009/*009 (7.3%). Furthermore, MICA*002/*008, MICA*004/*009, MICA*008/*008, and MICA*009/*057 had a frequency of 5.5% in the T1D patients’ group. Whereas the rest of the genotypes (the majority) observed in the T1D patients’ group had a frequency of 3.6% or below, which is probably due to the high genotypic diversity identified in the study group. Nevertheless, although the frequency of the MICA*009/*009 genotype was higher in the T1D patients’ group (7.3%) in comparison to the control group (3.9%), this difference did not reach statistical significance (*p* = 0.456). No significant differences in frequency among all examined genotypes were detected between the two studied groups.

Analysis of the STR polymorphism alleles (exon 5) of the *MICA* gene revealed only five different alleles in our Jordanian study group (healthy control and T1D patients); *A4, A5, A6, A9*, and *A5.1* (Table 5), while the remaining alleles which are considered rare alleles were not detected in our study sample [38,48]. Furthermore, *A6* allele (45.5%) was the most frequent *MICA* allele found in the T1D patients’ group, followed by *A5.1* (12.7%), *A9* (12.7%), *A5* (11.8%), and *A4* (9.1%). However, results showed no statistically significant differences in the frequency of all five STR alleles between the Jordanian T1D patients’ group and control group.

Fifteen different genotypes of exon 5 of the *MICA* gene among our study groups (control and T1D groups) were observed (Table 6). Genotypes with the highest frequency among the T1D patients’ group were *A6/A6* (25.5%), *A5.1/A6* (16.4%), and *A6/A9* (10.9%). However, no significant differences in frequency among genotypes between the control and T1D patients’ groups were detected.

## 4. Discussion

Considering *MICA* as a candidate gene for susceptibility to T1D is noteworthy because of its potential role in triggering autoimmune responses through its interaction with the NK2GD receptor [27,28,29], as well as its high level of polymorphism [21]. Data from studies performed on Italian [13], Korean [14], Belgian [15], Dutch [16], Spanish Basque [18], U.S. [39], African American [40], Nigerian [40], Australian [41], Slovenian [49], Japanese [50], Swedish [51,52], Latvian [53], and Chinese [54] populations examining the relation of *MICA* alleles and/or genotypes to T1D susceptibility revealed controversial results. However, to our best knowledge, this is the first study to examine the association of *MICA* polymorphisms with T1D in the Jordanian population. Polymorphisms in exons 2–4 as well as exon 5 (STR polymorphisms) of the *MICA* gene were analyzed in this study using a highly accurate genotyping method, namely the NGS method.

Data about the role of *MICA* gene polymorphisms in exons 2–4 in T1D pathogenesis remain sparse among populations. However, our results revealed no association between *MICA* polymorphisms and T1D susceptibility among Jordanian patients. Only two studies investigated their association with T1D, and our findings were similar to those reported by these studies performed on populations in the UK and Slovenia [42,49]. Nevertheless, the only significant difference between the control and T1D patient groups was for MICA*011 (*p* = 0.023), with a frequency of 6.9% in controls and a lower allelic frequency in T1D patients’ group (0.9%). On the contrary, most previous studies concentrated on studying the association of STR polymorphisms in exon 5 of the *MICA* gene with T1D and their findings were debatable. Regarding the *STR* polymorphisms in exon 5 of the *MICA* gene in our Jordanian T1D patients, our findings revealed no statistically significant differences in the frequency of all *STR* alleles between the Jordanian T1D patients’ group and control group. Our results are in accordance with the findings of studies performed in Australia [41], Finland [43], and the UK [42], which also showed no association between *STR* alleles and predisposition to T1D. On the contrary, studies performed in different populations identified different *STR* alleles as potential susceptibility factors for T1D development. The Korean and Japanese population identified *A4* as a risk and *A6* as a protective allele for T1D susceptibility [14,50]. On the other hand, a study performed in the Netherlands identified *A5* as a risk allele and *A6* as a protective allele for T1D [16]. Furthermore, studies performed in Sweden identified *A5* [51] and *A5.1* [52] as risk alleles and *A6* as a protective allele for T1D [51]. Moreover, studies in the Italian, Belgian, and Latvian populations identified *A5* as a risk factor for T1D development [13,15,53]. In addition, studies performed on Belgian as well as Spanish Basque volunteers identified *A9* as a protective allele against T1D [15,18] while another study performed on Chinese volunteers identified *A9* as a risk allele for T1D development [54]. Nevertheless, these controversial results refer to genetic diversity among different populations. The sample size of this study may be considered as a limitation. However, the number of patients included in this study represents all the T1D patients registered at the pediatric section at that the time of sample collection. We also acknowledge that another limitation of the study is the variation in gender distribution among the studied groups, but no studies regarding *MICA* allele association mentioned differences between males and females [13,14,15,16,40].

## 5. Conclusions

None of the *MICA* alleles and genotypes was observed to be positively associated with susceptibility to T1D in Jordanian patients. Nevertheless, only one negative association with T1D was detected in our population, namely the MICA*011 allele. Our study ascertains that different *MICA* alleles and genotypes seem to have no role in the pathogenesis of T1D in Jordanian patients. Nevertheless, analysis of additional candidate risk genes needs to be undertaken to clarify the contribution of these genes to the susceptibility to T1D. All in all, identifying possible risk genes might aid in developing an approach for screening these genetic markers associated with T1D in individuals in order to recognize individuals at risk for developing T1D. Nevertheless, early identification of individuals at risk of T1D development might help in prevention or at least delay of T1D disease onset.

## Figures and Tables

**Table 1:** Clinical parameters of the volunteers.

Parameters	Controls (*n* = 51)	T1D Patients (*n* = 55)
Age (years) (mean ± SD)	28.7 ± 6.5	20.2 ± 9.4
Male *n* (%)	32 (62.7%)	19 (34.5%)
Female *n* (%)	19 (37.3%)	36 (65.5%)
Age of disease onset (years) (mean ± SD)	-	10.1 ± 7.4

Type 1 diabetes (T1D).

**Table 2:** Primers utilized for amplifying exons 2–5 of the *MICA* gene.

Primer Name	Forward Primer	Reverse Primer
*MICA_1*	AAGGTTGGGACAGCAGACC	TTTCCCAGGACATCTTCTGCC
*MICA_2*	GGCAGAAATGCAGGGCAAAG	GCTCTCTGCCCCTAACTTTTCT
*MICA_4*	GCACTCAGCCCACACAGG	GTTTTGGGAGAGGAAGAGCT
*MICA_5*	CCAGGAGCTCCCAGCATTTC	AGATATCGCCGTAGTTCCTGC
*MICA_6*	ACCAAGACACACTATCACGCT	ATCAGGACACGATGTGCCAA
*MICA_9*	CCTGCCAGCCTGGAAGAAC	AAGCCCTGCATGTCACGG
*MICA_10*	TGCCCTTTCTTCTCCAGTGC	GTTCCATGTAGCAGGTGAACC
*MICA_11*	TGCCTGATGGGAATGGAACC	GACTCTGAAGCACCAGCACT
*MICA_exon5*	TGCTGGTGCTTCAGAGTCATT	TTACCATCTCCAGAAACTGCCCG

Major Histocompatibility Complex (MHC) class I chain-related gene A (*MICA*).

**Table 3:** Allele frequencies of exons 2–4 of the *MICA* gene among Jordanian T1D patients versus healthy controls.

Alleles (Exons 2–4)	Control (*n* = 102) *n* (%)	T1D (*n* = 110) *n* (%)	*p* Value	OR	95% CI
MICA*001	1 (1%)	0 (0%)	0.470	0.306	0.012–7.602
MICA*002	11 (10.8%)	15 (13.6%)	0.527	1.306	0.570–2.994
MICA*004	17 (16.7%)	12 (10.9%)	0.223	0.612	0.277–1.354
MICA*006	2 (2%)	1 (0.9%)	0.517	0.459	0.041–5.137
MICA*007	2 (2%)	0 (0%)	0.273	0.182	0.009–3.835
MICA*008	11 (10.8%)	15 (13.6%)	0.527	1.306	0.570–2.994
MICA*009	15 (14.7%)	26 (23.6%)	0.100	1.795	0.889–3.625
MICA*011	**7 (6.9%)**	**1 (0.9%)**	0.023 *	**0.125**	**0.015–1.030**
MICA*012	2 (2%)	0 (0%)	0.273	0.182	0.009–3.835
MICA*016	8 (7.8%)	5 (4.5%)	0.317	0.560	0.177–1.770
MICA*017	2 (2%)	0 (0%)	0.273	0.182	0.009–3.835
MICA*018	7 (6.9%)	9 (8.2%)	0.716	1.209	0.433–3.376
MICA*019	2 (2%)	2 (1.8%)	0.939	0.926	0.128–6.698
MICA*020	8 (7.8%)	9 (8.2%)	0.928	1.047	0.388–2.826
MICA*023	0 (0%)	1 (0.9%)	0.529	2.808	0.113–69.723
MICA*024	1 (1%)	2 (1.8%)	0.606	1.870	0.167–20.944
MICA*027	1 (1%)	3 (2.7%)	0.350	2.832	0.290–27.670
MICA*030	0 (0%)	1 (0.9%)	0.529	2.808	0.113–69.723
MICA*038	1 (1%)	0 (0%)	0.470	0.306	0.012–7.602
MICA*057	4 (3.6%)	4 (3.6%)	0.913	0.925	0.225–3.798
MICA*072	0 (0%)	1 (0.9%)	0.529	2.808	0.113–69.723
MICA*075	0 (0%)	1 (0.9%)	0.529	2.808	0.113–69.723
MICA*080	0 (0%)	1 (0.9%)	0.529	2.808	0.113–69.723
MICA*086	0 (0%)	1 (0.9%)	0.529	2.808	0.113–69.723

* Significant *p*-value ≤ 0.05.

**Table 4:** Genotype frequencies of exons 2–4 of the *MICA* gene among Jordanian T1D patients versus healthy controls.

Genotype (Exons 2–4)	Control (*n* = 51) *n* (%)	T1D (*n* = 55) *n* (%)	*p* Value	OR	95% CI
*001/*004	1 (2%)	0 (0%)	0.468	0.303	0.012–7.616
*002/*002	1 (2%)	1 (1.8%)	0.957	0.926	0.056–15.202
*002/*004	1 (2%)	2 (3.6%)	0.603	1.887	0.166–21.461
*002/*006	1 (2%)	1 (1.8%)	0.957	0.926	0.056–15.202
*002/*008	1 (2%)	3 (5.5%)	0.346	2.885	0.290–28.663
*002/*009	0 (0%)	2 (3.6%)	0.314	4.813	0.226–102.6
*002/*011	2 (3.9%)	0 (0%)	0.270	0.178	0.008–3.806
*002/*017	1 (2%)	0 (0%)	0.468	0.303	0.012–7.616
*002/*018	2 (3.9%)	1 (1.8%)	0.514	0.454	0.040–5.161
*002/*020	0 (0%)	2 (3.6%)	0.314	4.813	0.226–102.6
*002/*057	1 (2%)	1 (1.8%)	0.957	0.926	0.056–15.202
*002/*086	0 (0%)	1 (1.8%)	0.526	2.835	0.113–71.182
*004/*004	2 (3.9%)	2 (3.6%)	0.939	0.925	0.125–6.818
*004/*006	1 (2%)	0 (0%)	0.468	0.303	0.012–7.616
*004/*008	2 (3.9%)	1 (1.8%)	0.514	0.454	0.040–5.161
*004/*009	3 (5.9%)	3 (5.5%)	0.924	0.923	0.178–4.795
*004/*016	1 (2%)	0 (0%)	0.468	0.303	0.012–7.616
*004/*020	3 (5.9%)	1 (1.8%)	0.273	0.296	0.030–2.944
*004/*027	0 (0%)	1 (1.8%)	0.526	2.835	0.113–71.182
*007/*007	1 (2%)	0 (0%)	0.468	0.303	0.012–7.616
*008/*008	1 (2%)	3 (5.5%)	0.346	2.885	0.290–28.663
*008/*009	3 (5.9%)	2 (3.6%)	0.586	0.604	0.097–3.769
*008/*011	2 (3.9%)	0 (0%)	0.270	0.178	0.008–3.806
*008/*016	0 (0%)	2 (3.6%)	0.314	4.813	0.226–102.6
*008/*018	0 (0%)	1 (1.8%)	0.526	2.835	0.113–71.182
*009/*009	2 (3.9%)	4 (7.3%)	0.456	1.922	0.337–10.971
*009/*011	0 (0%)	1 (1.8%)	0.526	2.835	0.113–71.182
*009/*016	1 (2%)	0 (0%)	0.468	0.303	0.012–7.616
*009/*018	1 (2%)	1 (1.8%)	0.957	0.926	0.056–15.202
*009/*020	2 (3.9%)	2 (3.6%)	0.939	0.925	0.125–6.818
*009/*023	0 (0%)	1 (1.8%)	0.526	2.835	0.113–71.182
*009/*030	0 (0%)	1 (1.8%)	0.526	2.835	0.113–71.182
*009/*057	1 (2%)	3 (5.5%)	0.346	2.885	0.290–28.663
*009/*072	0 (0%)	1 (1.8%)	0.526	2.835	0.113–71.182
*011/*004	1 (2%)	0 (0%)	0.468	0.303	0.012–7.616
*011/*016	1 (2%)	0 (0%)	0.468	0.303	0.012–7.616
*011/*018	1 (2%)	0 (0%)	0.468	0.303	0.012–7.616
*012/*012	1 (2%)	0 (0%)	0.468	0.303	0.012–7.616
*016/*016	1 (2%)	1 (1.8%)	0.957	0.926	0.056–15.202
*016/*018	1 (2%)	1 (1.8%)	0.957	0.926	0.056–15.202
*016/*019	1 (2%)	0 (0%)	0.468	0.303	0.012–7.616
*016/*027	1 (2%)	0 (0%)	0.468	0.303	0.012–7.616
*017/*024	1 (2%)	0 (0%)	0.468	0.303	0.012–7.616
*018/*018	0 (0%)	1 (1.8%)	0.526	2.835	0.113–71.182
*018/*019	1 (2%)	0 (0%)	0.468	0.303	0.012–7.616
*018/*020	1 (2%)	0 (0%)	0.468	0.303	0.012–7.616
*018/*024	0 (0%)	2 (3.6%)	0.314	4.813	0.226–102.6
*018/*075	0 (0%)	1 (1.8%)	0.526	2.835	0.113–71.182
*019/*020	0 (0%)	1 (1.8%)	0.526	2.835	0.113–71.182
*019/*080	0 (0%)	1 (1.8%)	0.526	2.835	0.113–71.182
*020/*008	1 (2%)	0 (0%)	0.468	0.303	0.012–7.616
*020/*020	0 (0%)	1 (1.8%)	0.526	2.835	0.113–71.182
*020/*038	1 (2%)	0 (0%)	0.468	0.303	0.012–7.616
*027/*027	0 (0%)	1 (1.8%)	0.526	2.835	0.113–71.182
*057/*057	1 (2%)	0 (0%)	0.468	0.303	0.012–7.616

**Table 5:** Allele frequencies of exon 5 of the *MICA* gene among Jordanian T1D patients versus healthy controls.

Alleles (Exon 5)	Control (*n* = 102) *n* (%)	T1D (*n* = 110) *n* (%)	*p* Value	OR	95% CI
*A4*	10 (9.8%)	10 (9.1%)	0.895	0.920	0.366–2.311
*A5*	8 (7.8%)	13 (11.8%)	0.333	1.575	0.624–3.972
*A5.1*	16 (15.7%)	14 (12.7%)	0.537	0.784	0.361–1.700
*A6*	50 (49%)	50 (45.5%)	0.603	0.867	0.505–1.487
*A9*	16 (15.7%)	14 (12.7%)	0.537	0.784	0.361–1.700

**Table 6:** Genotype frequencies of exon 5 of the *MICA* gene among Jordanian T1D patients versus healthy controls.

Genotype (Exon 5)	Control (*n* = 51) *n* (%)	T1D (*n* = 55) *n* (%)	*p* Value	OR	95% CI
*A4/A4*	2 (3.9%)	1 (1.8%)	0.514	0.454	0.040–5.161
*A4/A5*	0 (0%)	1 (1.8%)	0.526	2.835	0.113–71.182
*A4/A5.1*	1 (2%)	3 (5.5%)	0.346	2.885	0.290–28.663
*A4/A6*	4 (7.8%)	2 (3.6%)	0.349	0.443	0.078–2.532
*A4/A9*	1 (2%)	2 (3.6%)	0.603	1.887	0.166–21.461
*A5/A5*	1 (2%)	2 (3.6%)	0.603	1.877	0.166–21.461
*A5/A5.1*	2 (3.9%)	4 (7.3%)	0.456	1.922	0.337–10.971
*A5/A6*	4 (7.8%)	4 (7.3%)	0.912	0.922	0.218–3.895
*A5/A9*	0 (0%)	1 (1.8%)	0.526	2.835	0.113–71.182
*A5.1/A5.1*	1 (2%)	2 (3.6%)	0.603	1.877	0.166–21.461
*A5.1/A6*	10 (19.6%)	9 (16.4%)	0.663	0.802	0.297–2.168
*A5.1/A9*	3 (5.9%)	3 (5.5%)	0.924	0.923	0.178–4.795
*A6/A6*	13 (25.5%)	14 (25.5%)	0.997	0.998	0.416–2.393
*A6/A9*	6 (11.8%)	6 (10.9%)	0.890	0.918	0.276–3.054
*A9/A9*	3 (5.9%)	1 (1.8%)	0.273	0.296	0.030–2.944

## Data Availability

The data are available from the corresponding authors upon request.
